# Noxic effects of polystyrene microparticles on murine macrophages and epithelial cells

**DOI:** 10.1038/s41598-021-95073-9

**Published:** 2021-08-03

**Authors:** Julia Rudolph, Matthias Völkl, Valérie Jérôme, Thomas Scheibel, Ruth Freitag

**Affiliations:** 1 Department of Biomaterials, Faculty of Engineering Sciences, University of Bayreuth, Bayreuth, Germany; 2 Department of Process Biotechnology, Faculty of Engineering Sciences, University of Bayreuth, Bayreuth, Germany; 3 Bayreuth Center for Colloids and Interfaces (BZKG), Universität Bayreuth, Bayreuth, Germany; 4 Bayreuth Center for Molecular Biosciences (BZMB), Universität Bayreuth, Bayreuth, Germany; 5 Bayreuth Center for Material Science (BayMAT), Universität Bayreuth, Bayreuth, Germany; 6 Bavarian Polymer Institute (BPI), Universität Bayreuth, Bayreuth, Germany

**Keywords:** Cell biology, Materials science

## Abstract

Microplastic (MP) contamination has been identified as an ecological problem with an increasing impact on everyday life. Yet, possible effects of MP at the cellular level are still poorly understood. Here, the interaction of murine macrophages (J774A.1, ImKC) and epithelial cells (STC-1, BNL CL.2) with well-characterized poly(styrene) MP particles (MPP) of varying sizes (0.2–6.0 µm) was studied. Macrophages are expected to actively engulf particles which could be confirmed in this study, while epithelial cells are found in tissues with direct contact with ingested or inhaled MPP. Here, the epithelial cells from both investigated cell lines did not ingest MPP in significant numbers. Concomitantly, no cytotoxic effects nor any influence on cellular proliferation were observed. Cells from the two macrophage cell lines showed high ingestion of MPP of all sizes, but cytotoxic effects were observed only for one of them (ImKC) and only at MPP concentrations above 250 µg/mL. Indications of cellular stress as well as effects on cell proliferation were observed for cell populations with high particle cell interactions.

## Introduction

Large-scale industrial plastic production started in the nineteen-fifties, initially using waste material from the chemical industry as the basis for the production of polyvinyl chloride (PVC)^1–3^. Low production costs, properties like durability, ductility, and lightweight have promoted the increasing use of plastic. Over eight billion tons of plastic have been produced since the beginning, and roughly 80% of the produced plastic is assumed to have accumulated in the environment ^4^. Over 10 million tons of plastic waste enter the oceans per year^5^, and the latest results indicate that the contamination of the terrestrial environment by plastics may be 4–23 times higher^6–8^. Due to (photo-)chemical, mechanical, and/or biological degradation, larger plastic residues tend to disintegrate into smaller particles^9^, so-called microplastic (MP) and nanoplastic. MP is defined as plastic fragments with a size between 0.1 µm and 5 mm^10^ and can today be found in all investigated environmental compartments^11–15^. MP has been shown to enter the food chain and to have an impact on the fitness of several species^16–19^.

One of the major microplastic entry points into organisms is the ingestion of contaminated food^20,21^. Ingested MPP then migrate through the gastrointestinal tract, where they may interact with the resident tissues and cause gut toxicity (e.g. inflammation of the gut lining). As a consequence, an impairment of the gut-vascular barrier can develop, and MPP then gain access to the liver via the portal vein^22,23^. Effects have in particular been shown in the presence of submicron and nanoparticles, i.e. particles with a diameter < 1 µm. In one study, polystyrene (PS) particles < 0.3 µm were found in the liver, spleen, blood, and bone marrow of rats after 10 days of feeding^24^, while particles with a diameter of 0.1 µm were uptaken with a 15- to 250-fold higher frequency by intestinal tissue compared to larger particles (≥ 0.5 µm)^25^.

Once foreign matter enters the body, among the first responders at the cellular level are cells of the immune system. Macrophages are specialized in engulfing foreign particular matter via phagocytosis^26^. In this context, exudate and resident macrophages can be differentiated^27^ Exudate macrophages are found in the bloodstream, patrolling the whole body ready to reach local inflammation sites^28^, while resident macrophages are confined to a specific tissue^29^. The latter are usually specialized in cell morphology and function^30^. Foreign particulate matter acts as a stimulus to activate macrophages. Macrophages, once activated, prime the immune system in various directions, which inter alia tags them as suitable test cells for cytotoxicity assays^31^. The response of macrophages to stimulation ranges from induced cell proliferation, secretion of reactive oxygen species (ROS)^32,33^ and interferon-α and -β^34^, to delayed hypersensitivity. Phagocytosis goes along with higher oxygen uptake and enhanced production of ROS, known as respiratory burst^35,36^. An increase in ROS resulting from MP ingestion has been already shown in vivo for different organisms^37,38^, however, cells grown in vitro seemed to be less affected^39,40^. At physiological levels, ROS function as “redox messengers” in intracellular signaling and regulation, whereas an excess of ROS induces cell death by promoting the intrinsic apoptotic pathway^41^.

Whereas phagocytosis is a mechanism for the uptake of larger particles (size ≥ 0.5 µm) and is observed only in specialized cell types like macrophages^42–45^, a second endocytotic uptake mechanism, namely pinocytosis can be performed by most cell types. Pinocytosis refers to the uptake of fluids or small particles (< 0.5 µm). Some cells can also take up larger particles (1–5 µm) by macropinocytosis^46^. For a given particle, the uptake mechanism depends on the particle size as well as on its interaction with (specific) receptors to the cellular membrane and in consequence on the cell type^42,44,47^. It has been shown that a given macrophage cell line uses different uptake mechanisms for particles of different sizes. For example, Geiser et al*.* showed that nanoparticles (0.078 µm in diameter) were ingested using nonphagocytic mechanisms, whereas 0.2 µm and 1 µm particles were ingested by phagocytic mechanisms^48^.

Direct effects of MMP on cells are considerably less well studied than their effects on the fitness of entire organisms. Olivier et al*.* observed cytotoxic effects on murine macrophages (J774A.1) and fibroblasts (L929) for 0.45 and 3.53 µm PS particles at concentrations above 500 µg/mL^49^. Hwang et al*.* identified cytotoxic effects of 3 µm PS particles at 1000 µg/mL on human dermal fibroblasts^50^. Stock et al. showed a reduced viability of Caco-2 cells, a human epithelial cell line, in the presence of 1 µm PS particles^51^. In most of these studies, MPP were mainly classified by polymer type, particle shape, and size^52–54^. A few studies emphasized the importance of the particle surface for cellular uptake and toxicity^55–58^. In this context, the so-called protein corona, i.e. the highly dynamic protein layer, which forms on the surface of any particle placed in a protein-containing solution, such as whole blood or plasma^59–61^, influences the physicochemical surface properties of MPP like surface charge and roughness^62,63^ and in turn, the cellular uptake^56,62–65^.

Although the particle size is widely recognized as decisive for cellular uptake, few comparative investigations using a series of MPP of a defined and narrow size distribution have been performed so far. Moreover, a clear understanding of how cells from the primary line of defense of the body (i.e. macrophages as part of the immune system and barrier-forming epithelial cells) react to exposure to MPP is largely lacking. In this work, we analyzed the impact of MPP on four murine model cell lines, two macrophage types (J774A.1 from ascites as an example for exudate macrophages and ImKC as an example for hepatic resident macrophages, i.e. Kupffer cells), one intestinal (STC-1) and one hepatic epithelial cell line (BNL Cl.2). Six batches of graded PS particles, each with narrow size distribution and covering a range from 0.2 to 6.0 µm were used. Besides uptake, the impact of the particles on cell metabolism and proliferation was investigated.

## Results and discussion

### ζ-Potential of MPP

The ζ-potential of MPP is assumed to influence cellular uptake^58,66,67^. Therefore, the ζ-potentials of all MPP were measured in 1 mM KCl (pH 6) and after incubation in the different growth media or fetal calf serum (FCS) diluted in DPBS (Table 1).

**Table 1:** ζ-potential analysis of differently sized PS-MPP. MPP were incubated in 1 mM KCl or pre-incubated overnight in growth media (DMEM or RPMI) or 10% (v/v) FCS in 1 × DPBS. The ζ-potential was measured in 1 mM KCl (pH 6). Data represent mean ± SD, n = 3.

MPP size (µm)	ζ-potential (mV)				
	KCl	10% FCS	RPMI 1640	DMEM_Lonza_	DMEM_ATCC_
0.2	− 47.4 ± 0.3	− 26.7 ± 0.3	− 26.3 ± 0.1	− 26.9 ± 0.2	− 26.0 ± 0.2
0.5	− 52.8 ± 0.2	− 25.2 ± 0.1	− 26.2 ± 0.1	− 25.2 ± 0.1	− 24.6 ± 0.2
1.0	− 66.1 ± 0.1	− 27.9 ± 0.1	− 29.2 ± 0.5	− 26.4 ± 0.0	− 25.0 ± 0.1
2.0	− 76.7 ± 0.3	− 29.9 ± 0.2	− 30.7 ± 0.1	− 29.3 ± 0.2	− 27.7 ± 0.4
3.0	− 79.0 ± 0.6	− 31.1 ± 0.3	− 29.6 ± 0.4	− 30.3 ± 0.2	− 28.6 ± 0.2
6.0	− 85.4 ± 1.4	− 10.1 ± 0.7	− 11.7 ± 0.2	− 12.7 ± 0.4	− 11.3 ± 0.3

The ζ-potential decreased with increasing MPP diameter from − 47.4 ± 0.3 mV for 0.2 µm particles to − 85.4 ± 1.4 mV for 6 µm particles in KCl. In growth medium and FCS, the ζ-potential was higher and the pronounced difference observed as a function of the particle size after incubation in KCl solution was not observed in this case. Instead, all ζ-potentials fell in the range between − 10 and − 30 mV. Similar effects of incubation in cell culture media on the ζ-potential have been observed previously^67,68^. Since the effect of FCS-containing culture media on the ζ-potential was similar to that of 10% FCS in DPBS, we assume that the observed effects are mainly due to a corona formed on the MPP by proteins from FCS rather than culture medium-specific components. Since all culture media used in our study contained FCS, we expect similar protein coronae for all MPP once they had come into contact with the cell culture media during the experiments. In consequence, all MPP are expected to show similar surface properties, and their reaction with different cell lines can be compared directly.

### MPP uptake by macrophages and epithelial cells depends on particle size

**Figure 1:**
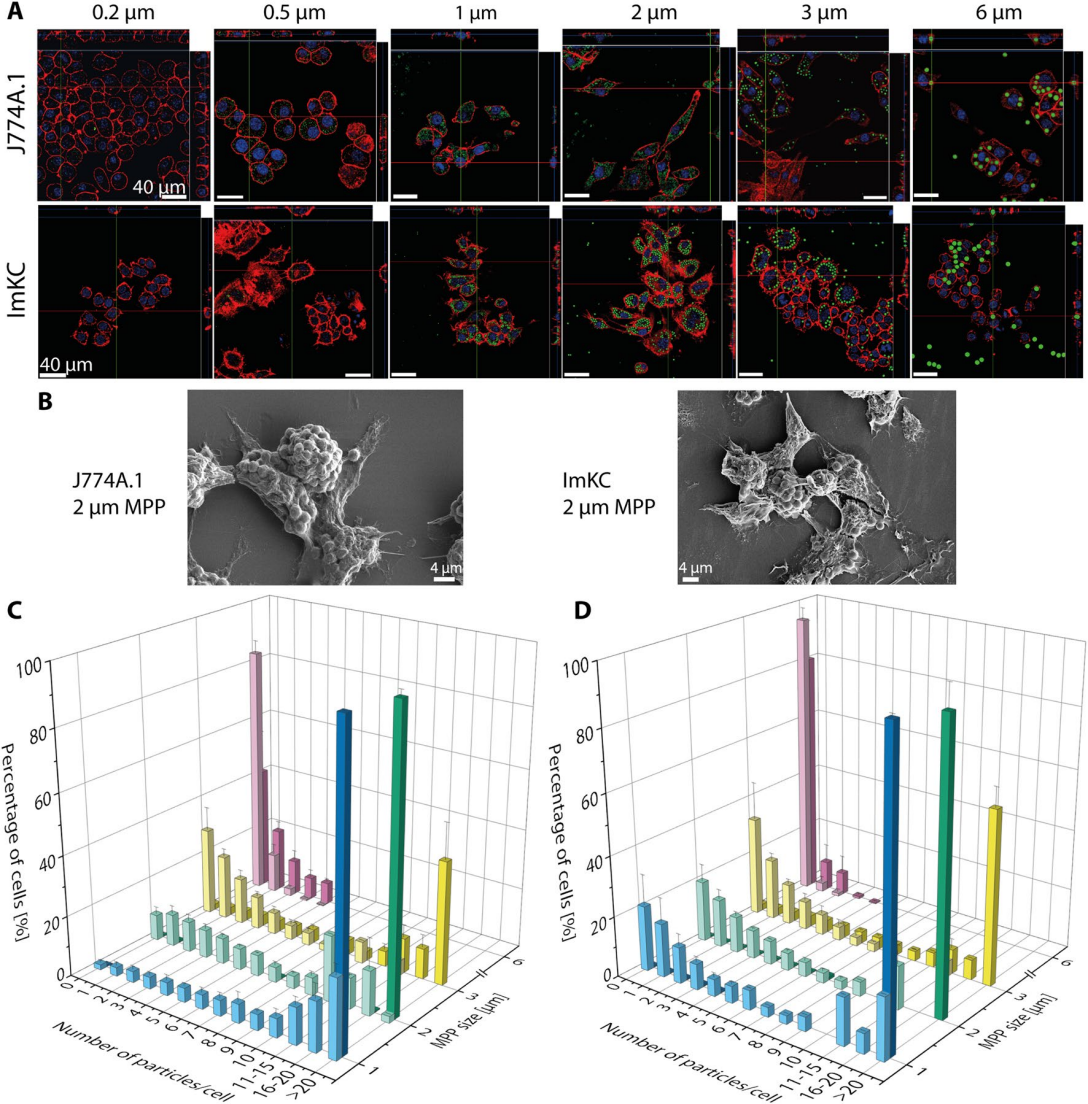
Analysis of size-dependent uptake of PS-MPP by J774A.1 and ImKC macrophages. Analysis was performed using confocal laser scanning microscopy (CLSM, **A**), scanning electron microscopy (SEM, **B**), and flow cytometry (**C**, **D**). (**A**) Size and number of added particles per cell: 0.2 µm: 750,000, 0.5 µm: 48,000, 1 µm: 2000, 2 µm: 700, 3 µm: 200, 6 µm: 25. Actin filaments were stained with rhodamine-phalloidin (red), nuclei were stained with DAPI (blue); FITC-fluorescent MPP are shown in green. Scale bars: 40 µm. (**B**) Representative SEM images are shown for each cell line in the presence of 2 µm MPP. Additional images are shown in Supplementary Fig. S2. Scale bars: 4 µm. (**C**,**D**) Results of flow cytometry measurements of J774A.1 (**C**) and ImKC (**D**). Shown are the percentage of cells in correspondence to the number of interacting particles per cell and the size of the particles. Light colors represent low concentration, dark colors the high concentration of MPP (see Table 2). Note that no data was available for 0.2 and 0.5 µm particles due to lacking resolution of the flow cytometer. The calculation of the number of interacting particles per cell is based on the fluorescence intensity of the particles (histograms, Supplementary Fig. S1). Data represent mean ± SD, n = 3.

MPP ingestion by the macrophage cells as a function of MPP size was analyzed using confocal laser scanning microscopy (CLSM) and scanning electron microscopy (SEM) (Fig. 1A,B, Supplementary Fig. S2). Flow cytometry was used to statistically quantify the interaction between MPP and macrophages (Fig. 1C,D) using two different concentrations (low concentration: lc, high concentration: hc, Table 2) of fluorescent PS-MPP (Supplementary Fig. S1).

**Table 2:** Overview of MPP concentrations used for proliferation tests, flow cytometry, and ROS assays. For all particle sizes, a low and high concentration, depending on the particle size, was applied. Low concentration =  < 1% coverage of the plate; medium concentration = 1–10% coverage of the plate; high concentration = 20–30% coverage of the plate.

Particle size	Low concentration (particles/cell)	High concentration (particles/cell)
0.2 µm	10	100,000
0.5 µm	10	10,000
1 µm	10	1000
2 µm	10	400
3 µm	10	100
6 µm	2	25

According to these data, ingestion of MPPs of all sizes was observed for both macrophage cell lines. J774A.1 cells seemed to ingest more MPP per cell than the ImKC cells. Using flow cytometry, in the case of the micron-sized particles, the numbers of MPP interacting per cell were obtained based on the fluorescence intensity (Supplementary Fig. S1). No direct quantification was possible for the submicron-sized particles (0.2 and 0.5 µm). However, in that case, a concentration depended shift was detected in fluorescence intensity for the whole cell population (Supplementary Fig. S1). For J774A.1 (Fig. 1C), high interaction rates were observed for MPP sizes between 1 and 3 µm. Increasing MPP concentrations resulted in a rising number of particle-cell interactions (PCI). In contrast, the majority of the J774A.1 cells did not interact with particles of 6 µm, and the 6 µm-MPP were in fact the only ones where no differences could be observed for low and high MPP concentrations. This effect may be related to the natural function of macrophages. Most air- and water-borne bacteria have a size between 1 and 4 µm^69^. Due to their “oversize”, 6 µm MPP become less attractive for ingestion. The ingestion of particles has in principle been reported for sizes up to 10 µm for J774A.1, but particle surface modifications with e.g. IgG or carboxyl groups were necessary in such cases, and ingestion rates were still rather low^43,68,70–72^. For the liver macrophage cell line ImKC, a similar trend as for J774A.1 was observed. The general tendency for particle uptake was lower, but a significant (p < 0.05) decrease in MPP-cell interactions was found only for 1 µm_lc_ MPP. As liver macrophages, ImKC cells are expected to be more specialized towards internalizing small particles^73,74^.

**Figure 2:**
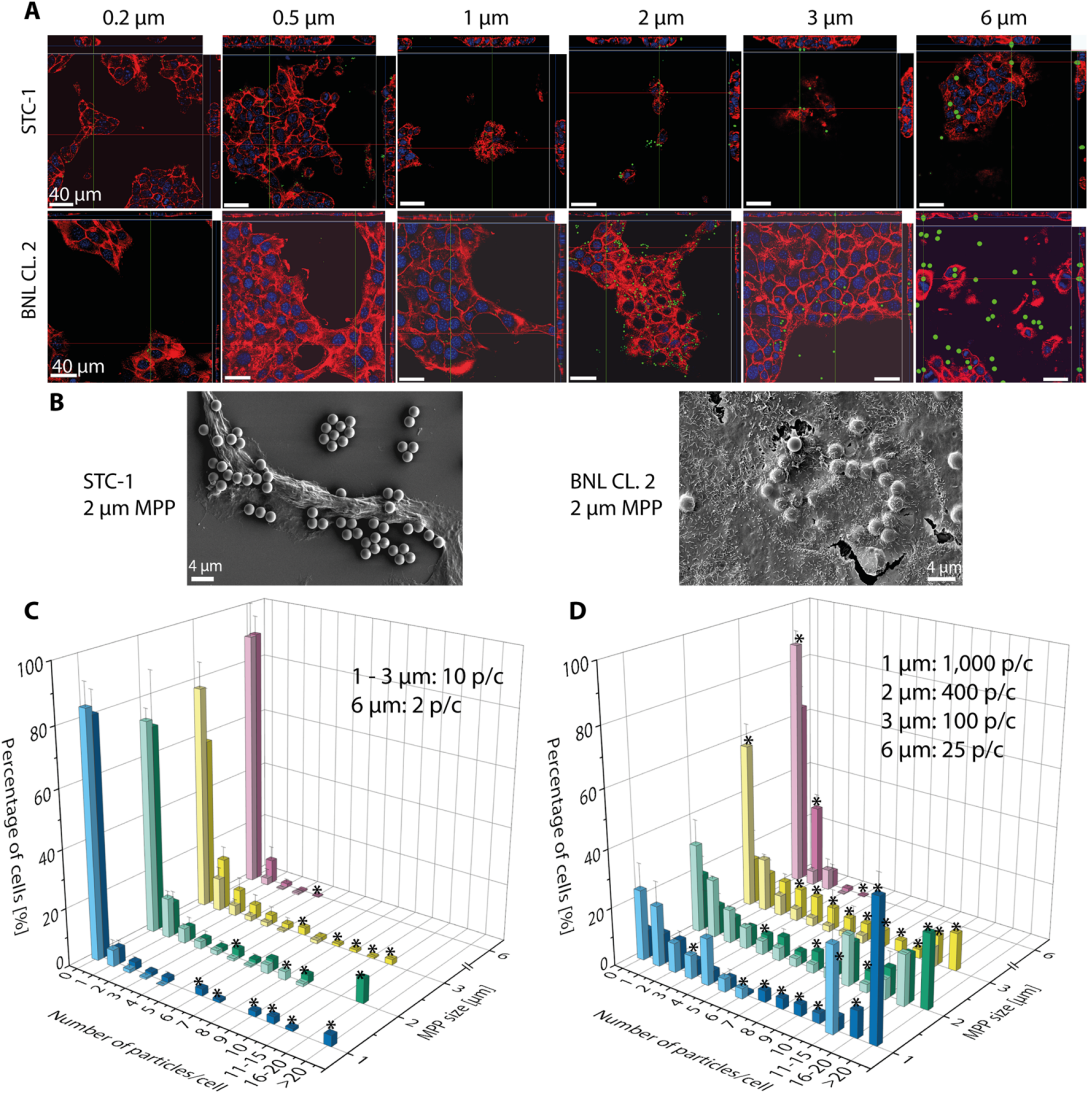
Analysis of the particle uptake by the epithelial cells (STC-1, BNL CL.2) as a function of size and concentration. Analysis was performed using confocal laser scanning microscopy (CLSM, **A**), scanning electron microscopy (SEM, **B**), and flow cytometry (**C**,**D**). (**A**) Size and number of added particles per cell: 0.2 µm: 750,000, 0.5 µm: 48,000, 1 µm: 2000, 2 µm: 700, 3 µm: 200, 6 µm: 25. Actin filaments were stained with rhodamine-phalloidin (red), nuclei were stained with DAPI (blue); FITC-fluorescent MPP are shown in green. Scale bars: 40 µm. (**B**) Representative SEM images are shown for each cell line in the presence of 2 µm MPP. Additional images are shown in Supplementary Fig. S2. Scale bars: 4 µm. (**C**,**D**) Results of flow cytometry measurements of STC-1 (light color) and BNL CL.2 (dark color) at low (**C**) and high (**D**) particle concentrations. The added number of particles per cell are given in the graph as p/c. No data are available for 0.2 and 0.5 µm particles due to lacking resolution of the flow cytometer. Data represent mean ± SD, n = 3. Stars represent statistically significant data points (p < 0.05) between both cell lines. For better clarity, no differentiation of significance level is shown.

In case of the intestinal epithelial cells (STC-1), no uptake of MPP larger than 0.2 µm was observed using CLSM and SEM (Fig. 2A,B, Supplementary Fig. S2). Larger MPP merely interacted with the cellular surface, which was detectable with both microscopic methods. This is corroborated by flow cytometry analysis (Fig. 2C,D). While there was no size effect, the MPP-cell-interactions increased slightly at higher concentrations. The lacking ingestion of particles above 0.2 µm and the low MPP interaction with STC-1 is consistent with previous studies focusing on nanoparticles, where particles with sizes of up to 0.12 µm were ingested by STC-1 cells via endocytosis^75,76^.

The hepatic epithelial cells (BNL CL.2) did take up MPP < 6 µm (Fig. 2A,B, and Supplementary Fig. S2), albeit at numbers smaller than the macrophages. BNL CL.2 cells have been described as non-phagocytotic^77,78^, but the ingestion of micron-sized particles could in principle also take place by macropinocytosis^42,46^. As recently described, hepatic epithelial cells can take up apoptotic or necrotic cells, since clearance of such cells is substantial to sustain tissue homeostasis^79^. Quantification of the interaction using flow cytometry data (Fig. 2D), corroborated again the CSLM and SEM measurements. At low concentrations, 1–3 µm-sized MPP, showed nearly no interaction with the cells. In contrast, MPP-cell interactions could be detected at high concentrations. In case of the 6 µm-MPP, almost no interactions took place with BNL CL.2 cells independent of the concentration. As shown in Fig. 2D, there are significant differences in the uptake behavior of the two cell lines. The intestinal epithelial cells (STC-1 cells) did not interact with particles even at high concentrations, while the liver epithelial cells (BNL CL.2 cells) showed internalization and higher interaction rates in particular at elevated MPP concentrations.

### Analysis of MPP effects on metabolic activity (MTT assay)

**Figure 3:**
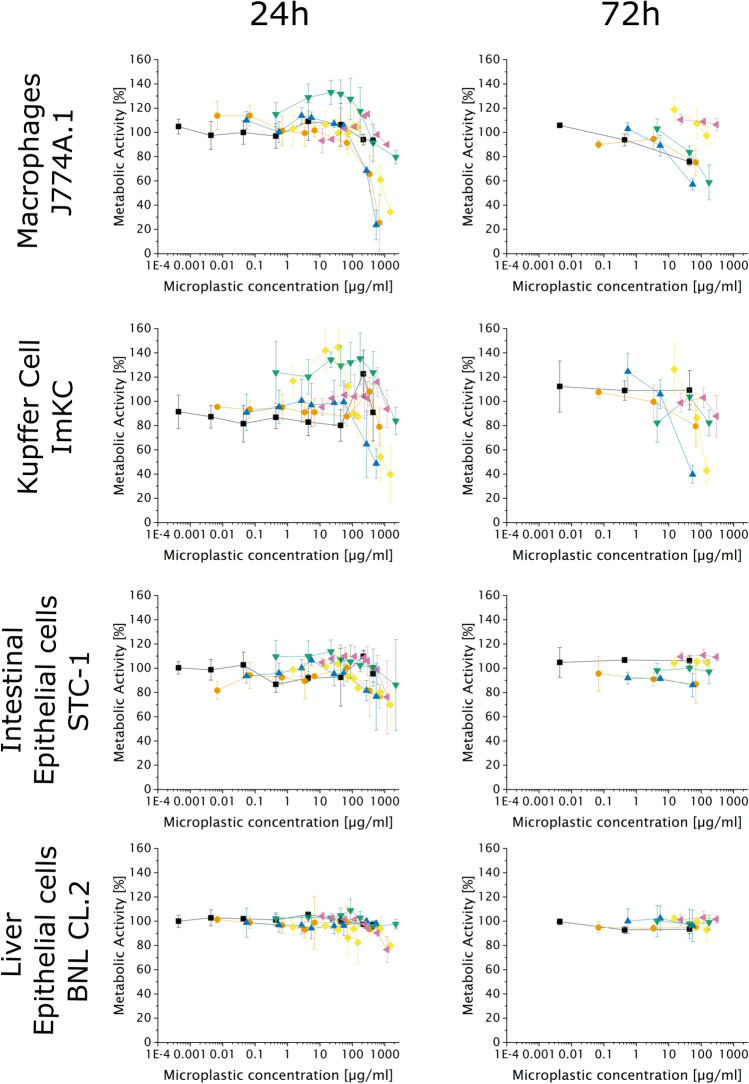
Cell metabolic activity after 24 h and 72 h in the presence of MPP. The metabolic activity was determined using the MTT assay in correlation to cells without particles acting as negative control. Data represent mean ± SD, n = 3 biological replicates. Particle sizes: black square = 0.2 µm, orange circle = 0.5 µm, blue triangle = 1 µm, green down-pointing triangle = 2 µm, yellow diamond = 3 µm, purple left-pointing triangle = 6 µm.

Uptake and accumulation of MPP in intracellular compartments could have a detrimental effect on cellular metabolism. The dose–response of macrophages and epithelial cells to MPP treatment was analyzed using an MTT assay (mitochondrial activity). Cells were incubated with increasing concentrations of 0.2–6 µm MPP, and the metabolic activity, i.e., MTT conversion by cellular oxidoreductases, was analyzed after 24 and 72 h of incubation (Fig. 3).

In epithelial cells, no negative metabolic effects were observed, no matter which concentration, MPP size, or incubation time was considered. This correlates well with the above-shown low tendency for MPP uptake. MPP merely attached to the cellular surface did not appear to affect cellular metabolism. Macrophages showed reduced metabolic activity, albeit only at MPP concentrations above 250 µg/mL after 24 h of incubation. These effects seemed to be related to the MPP size, since the reduction in metabolic activity was the highest for MPPs with 0.5–3 µm diameter, while 0.2 and 6 µm sized MPPs showed little to no effects over the concentration range tested. This result might be due to different uptake mechanisms for differently sized particles. Particles with diameters between 0.5 and 3 µm are mainly taken up via phagocytosis^55,80^, which is an energy-dependent process^81^, and this might explain the lower metabolic activity for cells with high phagocytic activity. In case of the 6 µm particles, on the other hand, we had previously observed no tendency for uptake. In consequence, a pronounced effect on the metabolic activity would have been surprising and was indeed not observed. Uptake of 0.2 µm sized particles seems to be part of a less energy-intensive uptake mechanism, which in consequence is less of a burden on the metabolism.

Noticeably, for 2 and 3 µm MPP in the low concentration range (10–37.5 µg/mL), we also detected a slight, but significant (p < 0.05) increase in metabolic activity (compared to non-treated cells) for ImKC cells. A similar trend was observed for J774A.1 cells after incubation with 2 µm MPP. This might be related to a hormetic response, which leads to a stimulation of the cellular metabolism in response to mild stress^82^. As recently published, such a response can be detected using the MTT assay^83^. An extension of the contact time between cells and particles (i.e., 72 h incubation) induces a drop in the metabolic activity already at concentrations of 10–100 µg/mL for both macrophage cell lines. ImKC seemed to be more sensitive than J774A.1.

Our results underline the importance of analyzing the size effects of MPP using preparations with narrow size distributions, as slight differences in size, e.g. between 0.2 and 0.5 µm, already lead to divergent metabolic responses. Our data further indicated the importance of MPP interaction/uptake, since both macrophage cell lines only showed a reduced metabolic response in the presence of particles between 0.5 and 3 µm, with high PCI.

### Effects of MPP size and concentration on the induction of intracellular ROS

**Figure 4:**
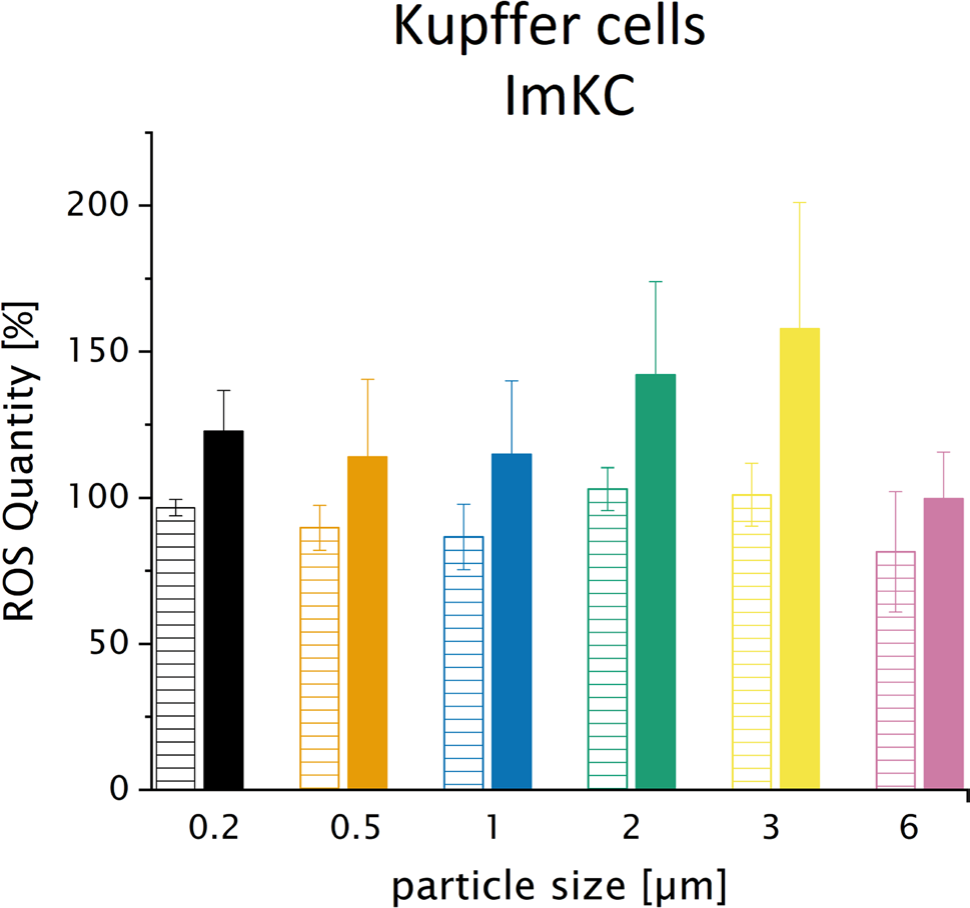
The appearance of reactive oxygen species upon increasing particle concentrations for ImKC macrophages. ROS of the whole cell population treated with an increasing particle concentration (striped bars = low concentration; full bars = high concentration). Quantity is represented in relation to a negative control without particles (100%). Data represent mean ± SD, n = 3.

ROS generation was analyzed after incubation of the cells with MPP of different sizes at low and high concentrations. For this purpose, the fluorescence resulting from oxidation of DCFDA by intracellular ROS was detected using flow cytometry (Fig. 4). In one out of four cell lines, namely ImKC, MPP concentration and size showed a tendency for higher ROS generation (Fig. 4), while the other three cell lines showed no enhanced ROS production (Supplementary Fig. S3). This finding is in line with work from other groups, also based on the study of entire cell populations in contact with MPP, i.e. these studies as well showed no statistically significant change in ROS production after MP treatment^39,40^. As an exception, the ImKC cells, i.e. cells from the resident liver macrophage cell line, showed a slight, but significant concentration-dependent rise in ROS. This increase was most pronounced in the presence of 1–3 µm MPP. It is known that Kupffer cells (i.e., resident macrophages) react quickly and non-specifically after phagocytosis of particular matter with an increase in ROS, while peritoneal and alveolar macrophages (i.e., exudate macrophages) react less strongly^84,85^.

**Figure 5:**
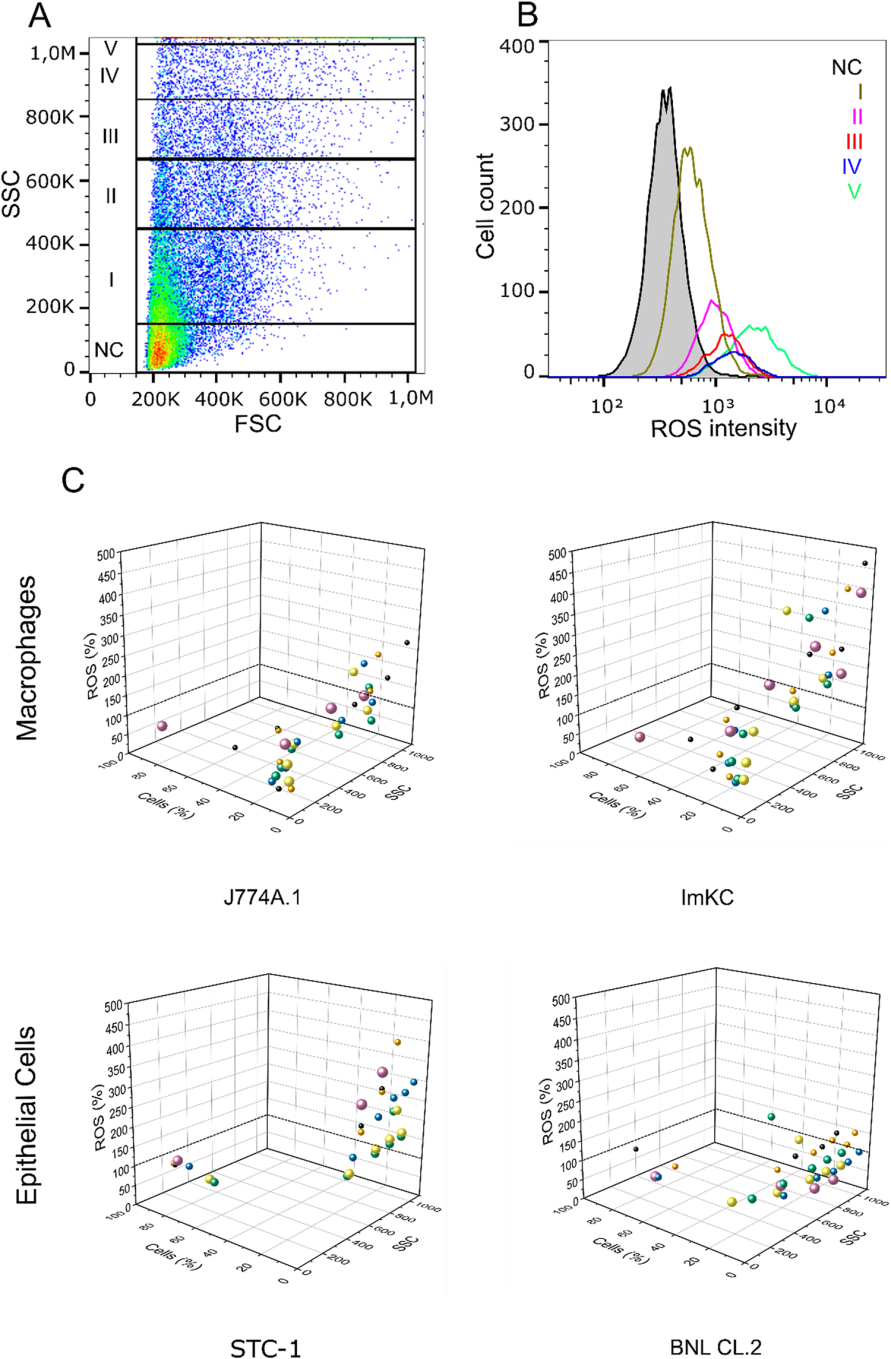
Correlation between ROS generation and intensity of cell-particle interaction. (**A**) Representative gating strategy for rising PCI. Gate NC represents cells with no particle interactions, I–V show increasing PCI (SSC = side scatter, FSC = forward scatter). (**B**) The respective ROS intensity for gated cells shown in (**A**). (**C**) Correlation between PCI, normalized ROS-quantity, and gated cell count. ROS quantity was normalized (control: cells incubated without particles). MFI is the mean SSC value of respectively gated cells. The dashed line highlights the relevant 100% ROS line, the back-ground ROS production determined in non-treated cells cultivated at otherwise similar conditions. Used particle concentration: h_c_ Table 2 (black circle = 0.2 µm, orange circle = 0.5 µm, blue circle = 1 µm, green circle = 2 µm, yellow circle = 3 µm, purple circle = 6 µm).

Flow cytometry was subsequently used to define cellular subpopulations. Here, it was necessary to use the SSC signal as a basis for PCI, since the fluorescence of the MPPs overlapped with the DCFDA fluorescence signal, which is why we had to use non-fluorescent particles (Fig. 5A). Either way, a higher SSC correlates with the higher fluorescence like higher fluorescence correlates with a higher particle count (Fig. 5B,C). The number of cells in each subpopulation was also taken into consideration for the interpretation of the results. In these experiments, a strong correlation was found between the level of MPP-cell-interaction and ROS response (Fig. 5B,C) for all cell lines, except the epithelial cell line BNL CL.2. However, the percentage of cells above the 100% ROS line (i.e., the ROS level in non-treated cells) corresponded only for ImKC cells to more than 35% of the entire population (Fig. 5C). Either way, an analysis of these small subpopulations might become important when analysing more complex systems or in vivo conditions, in which case cell–cell interactions get more important and small subpopulations might have a higher impact as expected.

For the other cells line, this fraction was much smaller. This explains why an increase in ROS was detectable for the entire ImKC population, while no effects could be observed for the other cell lines. STC-1 cells in particular showed a high ROS response in case of a high PCI, but only a few cells were concerned in their population. Moreover, on the level of the subpopulations, we also observed a decrease in ROS response (below 100% ROS) of cells with low PCI, especially among the macrophages. This response could be explained by an inflammatory response from cells with high PCI also present in the same culture. Such an inflammatory response includes the secretion of antioxidants and has previously only been described for cells challenged with nanoparticles^86^. A secretion of antioxidants would affect cells without or low MPP internalization and would result in a lower baseline ROS production compared to that of the negative control. Interestingly, the highest detected ROS responses were seen in case of small (0.2 and 0.5 µm) and large (6 µm) MPP for all cell lines, except for BNL CL.2. As mentioned in the introduction, small particles (< 0.5 µm) are known to be ROS-inducing. Different uptake mechanisms like phagocytosis for larger particles or less production of antioxidants due to lower uptake rates for the larger particles could explain the high ROS levels induced in the case of 6 µm particles.

### MPP effects on cell proliferation

**Figure 6:**
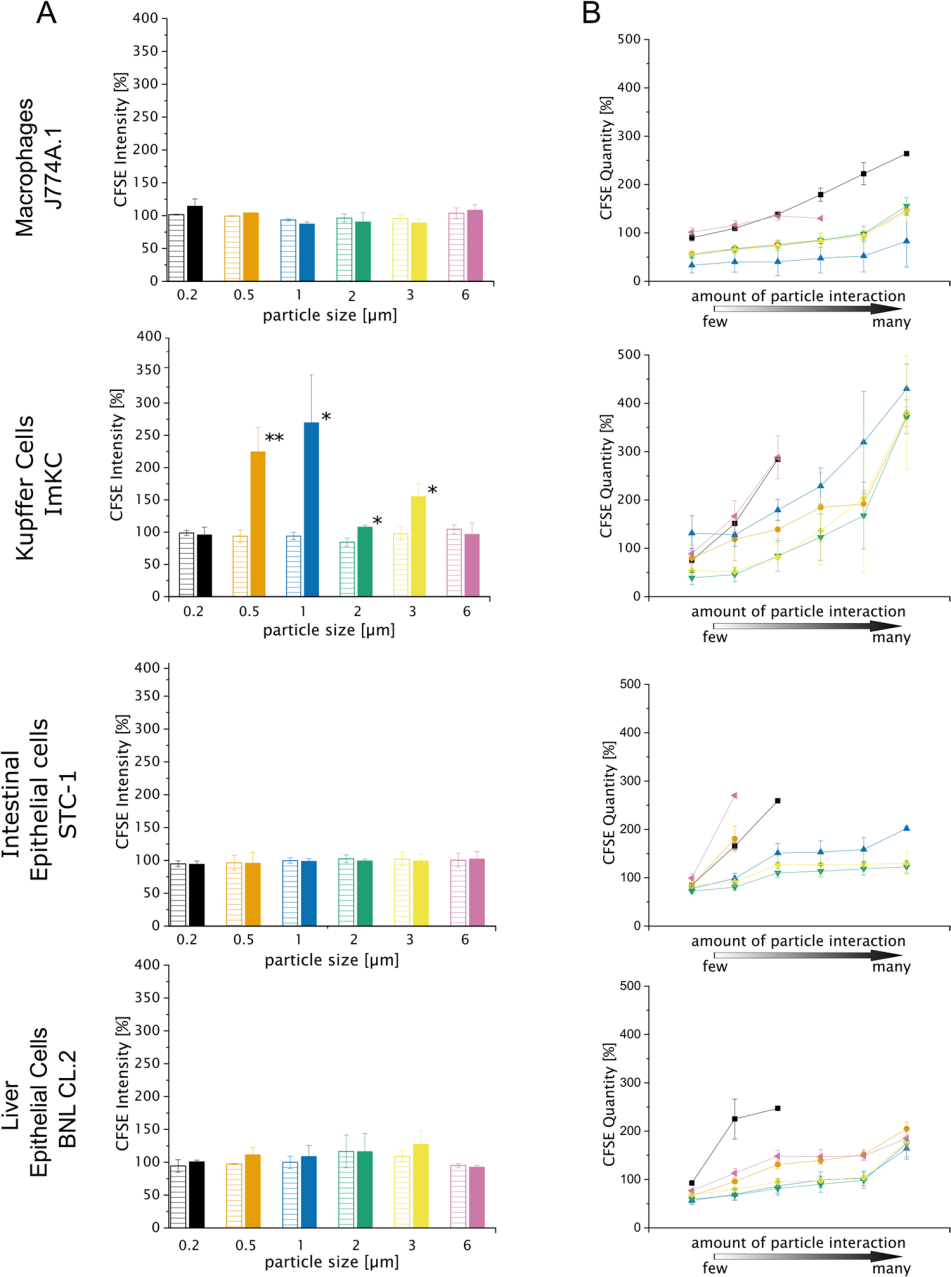
CFSE dilution assay after 72 h. CFSE assay was performed in presence of all particle sizes using all cell lines. Values were normalized (control: cells incubated without particles). As the CFSE intensity per cell is decreasing with every division, higher values for “CFSE intensity” indicate a reduced number of cell division. (**A**) Shows an analysis of the entire population while (**B**) adapts the same gating strategy for subpopulations as in Fig. 4A. Low MPP concentration: striped bars, high MPP concentration: filled bars. Black square = 0.2 µm, orange circle = 0.5 µm, blue triangle = 1 µm, green down-pointed triangle = 2 µm, yellow diamond = 3 µm, purple left-pointed triangle = 6 µm). Data represent mean ± SD, n = 3 biological replicates, *p < 0.05, **p < 0.01 compared to control.

To investigate the effects of MPP on cell proliferation, cells were incubated with MPP of all sizes at low and high concentrations (Table 2). First, resazurin assays were performed to examine the impact of MPP on proliferation at the entire population level. In that case, no significant effects were detected in any of the cell lines (Supplementary Fig. S4). As a result of this finding, we additionally performed a CFSE assay, since effects might only be seen at the subpopulation level. For ImKC cells, proliferation was significantly retarded in presence of particles between 0.5 and 3 µm, when the entire population was considered (“nonapoptotic cells” gate, Fig. 6A). To estimate the MPP effects on cell proliferation at the sub-population level, CFSE dilution assays and flow cytometry analysis were combined, while applying the previously discussed gating strategy (Fig. 5). When analyzing these subpopulations, it became clear that cells with higher PCI also displayed a higher CFSE intensity, indicative of a lower number of cell divisions (Fig. 6B). The correlation between the PCI and the observed effect on proliferation was not as pronounced as for the ROS data, but still statistically significant (p < 0.05). As for the ROS assay, these subtle differences between subpopulations were lost when the population was studied as a whole.

The correlation between high PCI and high CFSE is intriguing. It may simply be due to a dilutive effect, i.e. as cells divided, they dilute both the attached/ingested particles and the concentration of the CFSE labeled protein. However, cells with high PCI also tended to show increased ROS production in the above experiment, and increased levels of ROS have been shown to exert an effect on cell proliferation. Oxidative stress induced by microplastic might lead to DNA damage^32^. Hence, the accumulation of ROS in cells displaying high PCI might also influence cell division. A decrease in cell proliferation, has been shown already for high ROS levels in cancer cells^87^. A decrease in cell division, as seen in the CFSE data, correlates well with the highest ROS accumulation detected in the ImKC cells.

Another possible explanation of the effect of a high PCI on proliferation may again rely on the uptake mechanism. Macrophages need to spend energy on phagocytosing particles. Therefore, less energy is available for cell division. This dynamic energy budget model has been developed for multicellular organisms and was shown recently to be applicable for bacteria as well^88^.

## Conclusion

Polystyrene microplastic particles (MPP) are well uptaken in the case of exudate and resident macrophages (Kupffer cells) and much less in the case of hepatic and intestinal epithelial cells. Uptake and interaction rates were MPP-size as well as cell-type dependent. Such variations are in line with the cell phenotype. Macrophages are scavenger cells per-se programmed to take-up particulate matter, whereas the material uptake in epithelial cells is mostly limited to molecular transports. Despite the high uptake by the macrophages, a decrease in metabolic activity could only be measured at very high MPP concentrations, while no negative effects were observed for epithelial cells. At the subpopulation level, high particle-cell interactions/uptake tended to correlate with an overproduction of ROS, as well as with a reduction of the proliferative capability for all cell types. Altogether, we can conclude that scavenger cells are more susceptible to noxic effects of MPP ingestion, which are highly correlated to the number of MPP found in the cells. While there is little evidence of acute toxicity caused by the MPP, chronic toxicity due to intracellular accumulation of MPP cannot be excluded at this point and will be clarified by long-term studies. Our results also demonstrate that considering only the whole cellular population for analysis of MPP effects might bias the final results, as correlations involving only small cellular subpopulations may be masked. Hence, for investigations concerning cellular effect of microplastics, there is an urgent need to perform analysis at cell subpopulations or even better at the single-cell level.

## Materials and methods

### Materials

If not otherwise indicated, Greiner Bio-One (Frickenhausen, Germany) and Thermo Fisher Scientific (Schwerte, Germany) were used as suppliers for cell culture materials. Penicillin, streptomycin, Dulbecco’s Phosphate-Buffered Saline without Ca^2+^ and Mg^2+^ (DPBS), RPMI1640 (Roswell Park Memorial Institute), and DMEM_Lonza_ (Dulbecco`s Modified Eagle’s Medium; 3.7 g/L NaHCO_3_, l-glutamine-free) were obtained from Lonza (Lonza Group Ltd, Basel, Switzerland). DMEM_ATCC_ (1.5 g/L NaHCO_3_, 0.11 mM Na pyruvate, 4 mM l-glutamine) was obtained from ATCC (ATCC LGC Standards GmbH, Wesel, Germany). Modified Eagle Medium without phenol red (MEM) was obtained from Thermo Fisher Scientific (Schwerte, Germany). Fetal calf serum (FCS) was purchased from Sigma Aldrich (Taufkirchen, Germany). Based on the respective standard cell growth media, “conditioned media” were derived as follows: the respective culture supernatant was recovered after 24 h incubation with cells and sterile-filtered using a 0.2 µm cellulose acetate filter before supplementation with 2 mM glutamine. Conditioned media were stored at 4 °C until further use.

Phalloidin-tetramethylrhodamine B isothiocyanate, DAPI, 3-(4,5-dimethyl-2-thiazolyl)-2,5-Diphenyl-2H-tetrazolium bromide (MTT), 2′,7′-dichlorofluorescein diacetate (DCFDA), antimycin A from *Streptomyces* sp., and carboxyfluorescein succinimidyl ester (CFSE) were obtained from Sigma Aldrich (Taufkirchen, Germany). AlamarBlue (CellTiter-Blue Cell Viability Assay) was purchased from Promega (Walldorf, Germany).

Non-functionalized (plain) non-fluorescent and fluorescent polystyrene particles (Yellow Green, PS-YG) were obtained from Polysciences (Polysciences Europe GmbH, Eppenheim, Germany) with the parameter as follows: diameter of 0.2 µm (Cat. # 07304-15 (non-fluorescent), 17151-10 (fluorescent), 5.68 × 10^12^ particles/mL, size coefficient of variation (CV) ≤ 8%), 0.5 µm (Cat. # 07307-15 (non-fluorescent), 17152-10 (fluorescent), 3.64 × 10^11^ particles/mL, size CV ≤ 3%), 1 µm (Cat. # 07310-15 (non-fluorescent), 17154-10 (fluorescent), 4.55 × 10^10^ particles/mL, size CV ≤ 3%), 2 µm (Cat. # 19814-15 (non-fluorescent), 18338-5 (fluorescent), 5.68 × 10^9^ particles/mL, size CV ≤ 5%), 3 µm (Cat. # 17134-15 (non-fluorescent), 17155-2 (fluorescent), 1.68 × 10^9^ particles/mL, size CV ≤ 5%) and 6 µm (Cat. # 07312-5 (non-fluorescent), 17156-2 (fluorescent), 2.10 × 10^8^ particles/mL, size CV ≤ 10%). All MPP were delivered as a sterile aqueous suspension with a concentration of 2.5% (w/v). According to the supplier, all MPP are plain particles with little anionic charge due to residues of sulphate ester groups. Non-fluorescent particles showed no autofluorescence in the ex/em ranges of interest for the intended experiments. Prior to use, MPP stock solutions were diluted to the desired concentration in the respective growth media.

### Cell culture

Murine cell lines: Macrophages J774A.1 [from ascites, TIB-67, population doubling time: 17 h (according to supplier information)], intestinal epithelial-like cells STC-1 (CRL­3254, population doubling time: 54 h^89^) and hepatic epithelial cells BNL CL.2 [TIB-73, population doubling time: 40 h (according to supplier information)] were obtained from the American Type Culture Collection (ATCC, Manassas, USA). The hepatic macrophage cell line ImKC (Kupffer cells, SCC119, population doubling time: 24 h^73^) was obtained from Merck (Merck KGaA, Darmstadt, Deutschland). ImKC cells were cultivated in RPMI1640 supplemented with 2 mM glutamine. STC-1, BNL CL.2, and J774A.1 cells were cultivated in DMEM (DMEM_ATCC_ for STC-1 and BNL CL.2; DMEM_Lonza_ for J774A.1). For J774A.1 cells, the medium was additionally supplemented with 4 mM glutamine, 24 mM HEPES, and 0.1 mM sodium pyruvate. All media were supplemented with 10% (v/v) FCS and 100 U/mL penicillin/streptomycin, and are referred to as “growth media” throughout the manuscript. The cells were cultivated in a standard cell culture incubator (5% CO_2_/95% humidity) at 37 °C. For cell maintenance, all cell lines were passaged three times a week at a starting concentration of about 100,000 cells/mL. For detaching cells, either at 37 °C pre-warmed citric saline buffer (135 mM potassium chloride—15 mM sodium citrate, 5 and 10 min incubation at 37 °C for J774A.1 and ImKC, respectively) or 1 × Trypsin/EDTA (for STC-1 and BNL CL.2) was used.

### ζ-Potential measurement

The ζ-potential measurements were performed using the LiteSizer 500 (Anton Paar Germany GmbH, Ostfildern-Scharnhausen, Germany) and Omega cuvettes (Anton Paar Germany GmbH, Ostfildern-Scharnhausen, Germany). For ζ-potential measurements, 2.5 µL of the particle solutions were directly diluted in 1 mL of a 1 mM aqueous KCl solution (pH 6) and measured immediately. In some cases, 2.5 µL of the 25 mg/mL particle solutions were incubated in 1 mL growth medium (DMEM_ATCC_, DMEM_Lonza,_ and RPMI1640) or a mixture of 10% (v/v) FCS in DPBS overnight at 37 °C. Thereafter, the particles were collected by centrifugation [17,000*g* for 40 min at room temperature (RT)] and resuspended in 1 mL of the 1 mM KCl solution for measurement. Three measurements with at least 100 runs each were performed at 21 °C with an adjusted voltage of 200 V. The ζ-potential was calculated using the Helmholtz-Smoluchowski equation^90^.

### Flow cytometry

Flow cytometry analyses were performed using a CytoFLEX S or a Cytomics FC500 (Beckman Coulter, Krefeld, Germany). Both devices are equipped with a 488 nm laser. For each sample, at least 30,000 events were measured. For analysis, cells were washed twice with DPBS, detached by trypsinization or by citric acid treatment as described above, and suspended in 1 mL culture medium. Cells were then recovered by centrifugation (200*g*, 5 min), the supernatant was discarded and the cell pellet was resuspended in 0.5–1 mL DPBS. Forward scatter (FSC), side scatter (SSC), and FITC fluorescence (525 nm filter) were recorded. Cells were initially evaluated by scatter properties (FSC/SSC) to select a region (“nonapoptotic cell” gate) representing single, nonapoptotic cells while disregarding debris, and cellular aggregates. Upon uptake or cellular interactions of MPP, the SSC-fluorescence increased, and defined sub-populations become visible in most cases. Taking advantage thereof, we defined additional sub-gates to analyze the response of the sub-populations to non-labeled MPP in terms of production of ROS (ROS assay, DCF fluorescence) and influence on cellular proliferation (CFSE-dilution assay, CFSE fluorescence). Using labeled MPP (PS-YG), the number of particles was also quantified interacting with the cells.

The quantification of microparticles (1–6 µm) is based on the median fluorescence intensity of the particles. The fluorescence intensity of the microparticles was determined in the absence of cells, and this value was assumed to be the fluorescence intensity of one MPP on average. Due to the linear relation of the fluorescence intensity and the number of MPPs (for a plot of the correlation see Supplementary Fig. S1), it was possible to quantify particle numbers per cell. It should be noted that a differentiation between particle uptake and mere particle adhesion to the cells was not possible using flow cytometry. For sub-micron sized particles, no correlation was detectable, since the resolution of fluorescence difference between single particles was too low.

Flow cytometry data were evaluated using FlowJo software v 10.5.0 (Tree Star, Stanford University, CA, USA, 2018).

For ROS assay, both concentrations of non-fluorescent MPPs do not reveal any fluorescence above the autofluorescence of the cells in the DCF channel (Em. 526 nm).

### Qualitative analysis of MPP uptake

For the qualitative analysis of the ingestion of the particles by the cells, 15,000 cells were seeded in each well of an 8-well Ibidi slide (µ-Slide 8 Well, ibiTreat, ibidi GmBH, Gräfelfing, Germany). After 24 h in a cell culture incubator, cells were incubated with fluorescent particles (MPP per cell: 0.2 µm: 750,000, 0.5 µm: 48,000, 1 µm: 2000, 2 µm: 700, 3 µm: 200, 6 µm: 25) (total cultivation volume: 300 µL). Thereafter, cells were fixed for 15 min at RT with pre-heated 3.7% (v/v) paraformaldehyde in 1 × DPBS. Afterward, the cells were permeabilized with 0.1% (v/v) TritonX-100 for 10 min at RT. Actin filaments and nuclei were stained with 100 nM rhodamine-phalloidin and 100 nM DAPI, respectively. The samples were analyzed using a confocal laser scanning microscope (TCS SP8, 63 × oil immersion objective, laser: 408 nm, 488 nm, and 552 nm, Leica Microsystems, Wetzlar, Germany). Z-stacks were taken with a step size of 0.2–0.33 µm.

The MPP uptake was also analyzed using scanning electron microscopy (SEM). Therefore, 100,000 cells per slide were seeded on Ø 13 mm Nunc™ Thermanox™ slides (Thermo Fisher Scientific, Waltham, MA, USA) and incubated for 24 h to allow for cell adhesion (total cultivation volume: 120 µL). Then, 5 µL of a 180 mg/L non-fluorescent particle solution were added to the cells, which corresponds to 20 particles per cell. After another 24 h incubation, the cells were directly fixed using Karnovsky’s reagent (4% (v/v) formaldehyde, 5% (v/v) glutaraldehyde, with a final concentration of 32 mM PBS, pH 7.4) for 1 h at RT and afterward dehydrated using an ethanol series 50%, 70%, 80% for 30 min, 90% and absolute ethanol for 1 h. The overnight air-dried samples were sputter-coated with gold, and images were obtained using SEM [FEI Apreo Volumescope, Thermo Fisher Scientific, magnification: 10,000×, 2 kV, Everhart–Thornley detector (ETD)].

### Quantitative analysis of MPP uptake

**Scheme 1:**
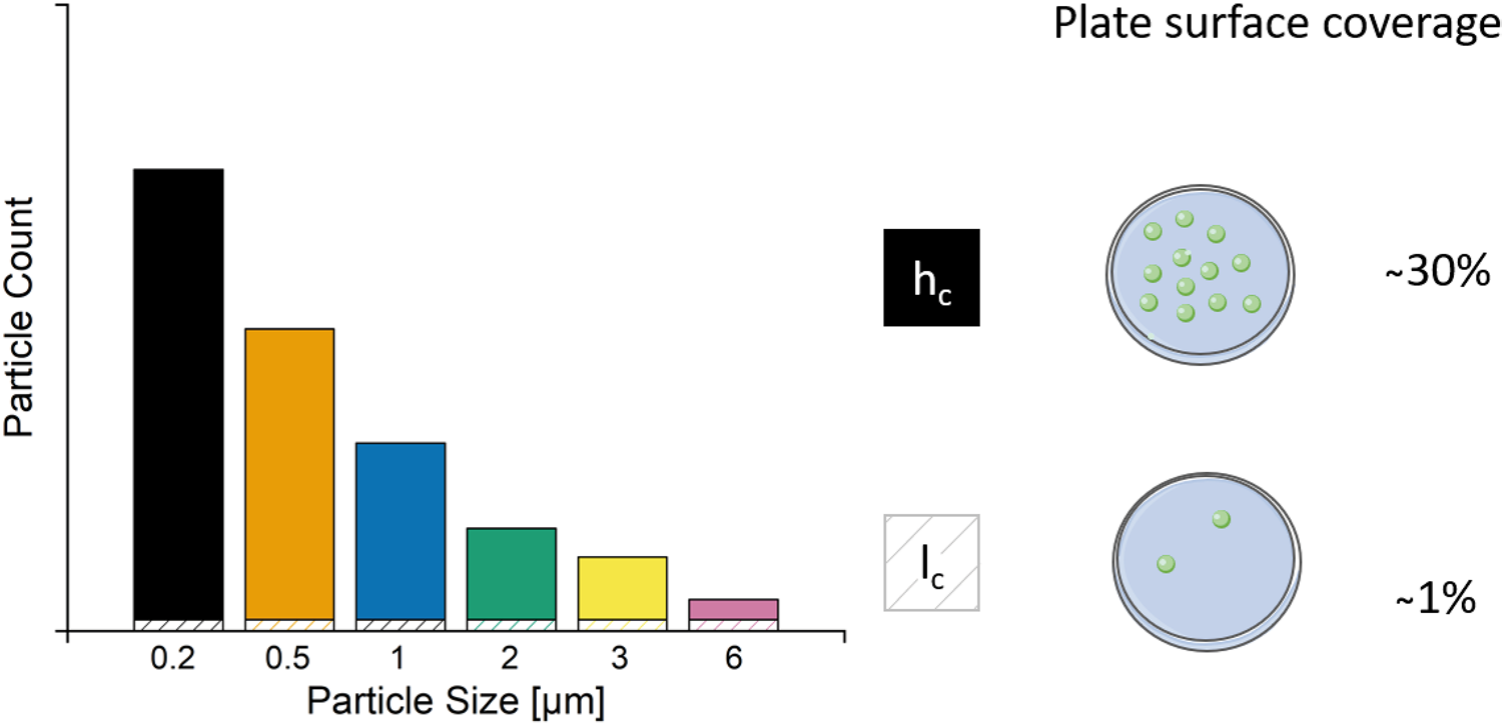
Relative particle count for low (l_c_) and high (h_c_) concentration for the respective particle size and the surface coverage per well. Specific values are given in Table 2.

For quantification of the particle-cell-interaction, 700,000 cells per well were seeded in 6-well culture plates and incubated for 24 h for cell adhesion. Afterward, fluorescent particles of various sizes were added at two concentrations, low and high, to the cells. Here, the low and high concentrations range from 2 to 10 MPP (low) and from 25 to 100,000 per cell (high), respectively, depending on the MPP size (Scheme 1, Table 2). Particle concentration was scaled in a logarithmic manner for the smallest particle sizes (0.2, 0.5 µm). For the bigger particles, the added number of particles was roughly scaled to correspond in volume to that added in case of the smaller particles, since otherwise the cells would have been overcrowded. A maximum concentration of 100,000 particles per cell was added in case of 0.2 µm sized particles compared to roughly 100 particles in case of 6 µm ones. After another 24 h of incubation, the cells were collected as described above and analyzed using flow cytometry.

### MTT assay

The influence of MPP on the metabolic activity of the cells was analyzed using an MTT assay. This tetrazolium salt-based cell viability assay is a recognized method for the toxicological assessment of PS microparticles^50,73,91^. Briefly, cells were seeded at 10,000 cells/well in 96-well plates (100 µL medium per well). For STC-1 cells, the seeding cell density was increased to 25,000 per well to accommodate for the slower growth rate and metabolism. After 24 h of incubation, the medium was aspirated, and 100 µL of the freshly prepared particle suspension was added. For this purpose, the desired particle concentrations were prepared by diluting the particle stock solution with the respective growth medium. The cells were then incubated for another 24 h. In case of the experiments with 72 h incubation time, the seeding density was reduced, i.e. adapted to the different cellular growth rates, and “conditioned medium” was used. The seeding densities, in this case, were 2000 cells/well (ImKC and BNL CL.2), 4000 cells/well (J774A.1), and 6000 cells/well (STC-1). After 24 h of incubation, the medium was aspirated, and 100 µL of freshly prepared particle dilutions were added for 72 h. After the incubation with the MPP, the medium was aspirated, cells were then washed with DPBS, and 50 µL freshly prepared MTT reagent (1 mg/mL MTT in MEM) was added to each well. After 2 h incubation, the supernatant was removed, and 100 µL of isopropanol were added per well to dissolve the produced formazan crystals. After 5 min shaking at 600 rpm, the absorbance at 570 nm (reference wavelength 650 nm) was measured using a TECAN GENios Pro plate reader (Tecan Austria GmbH, Gröding). Cells incubated without particles or with 0.3% Triton X-100 in the respective cell culture medium, under otherwise identical conditions, were used as negative and positive controls, respectively.

### Reactive oxygen species (ROS) assay

Intracellular ROS can be labeled with the non-fluorescent membrane-permeable dye DCFDA^92^, which is converted into fluorescent 2′,7′-dichlorofluorescein (DCF) upon oxidation by intracellular ROS. For analysis, 150,000 cells/well were seeded in 12-well plates in 1 mL of the respective culture medium. After 24 h of incubation, the indicated amounts of the freshly prepared particle suspensions (low and high concentration, Scheme 1, Table 2) were added and the plates were further incubated. Cells incubated without particles or in the presence of 50 µM (5 µM for STC-1) antimycin A under otherwise identical conditions were used as negative and positive controls, respectively. After 60 min incubation, 37.5 µM DCFDA was added per well (5 µM for STC-1), followed by another 24 h incubation. For STC-1 cells, the DCFDA concentration was reduced because of concerns regarding the cytotoxicity of the dye, which had manifested itself during the establishment of the assay. Afterward, the DCF fluorescence intensity was measured using flow cytometry.

### Resazurin proliferation assay

Resazurin is converted into resorufin, a highly fluorescent dye, by a reduction reaction in the mitochondria. Cells were seeded (15,000 cells for J774A.1 and BNL CL.2; 20,000 cells for ImKC and STC-1, three technical replicates) in 48-well plates. After 24 h of incubation, freshly prepared particle suspensions (20 µL) were added (“treated” cells), final concentrations are given in Table 2 (low and high concentrations). Cells incubated without particles under otherwise identical conditions were used as control (“non-treated” cells). After an additional 24 h incubation, the cell culture medium was removed and 350 µL of a 10% (v/v in the respective cell culture medium) AlamarBlue solution was added. Samples were incubated for 2.5 h. To estimate the background fluorescence of the AlamarBlue solution (F_Blank_), three wells without cells and particles (i.e., exclusively containing the AlamarBlue solution) were incubated. After incubation, aliquots of the cell culture medium (100 µL) were collected, and the resorufin fluorescence (Ex. 530 nm/Em. 600 nm) was analyzed using a plate reader (Mithras, Berthold Technologies, Bad Wildbad, Germany). The remaining AlamarBlue solution was removed from the wells and fresh cell culture medium was added. After two additional cultivation days, an AlamarBlue assay was performed as described above. For the statistical analysis, the mean value of the AlamarBlue control (F_blank_) was subtracted from each value of the same well plate. For the determination of the change in fluorescence per hour representing cell proliferation, the fluorescence intensity as detected on day three was subtracted from that of day one (Eq. 1),

1 $${\Delta }_{Fluorescence \; intensity/hour}=\frac{({F}_{sample} d3-{F}_{blank} d3)-({F}_{sample} d1-{F}_{blank} d1)}{48 h}$$

with F_sample_ being the fluorescence of the sample (either F_treated_ or F_non-treated_), F_blank_ being the fluorescence of the AlamarBlue control without cells, d3 being the incubation time of three days and d1 the incubation time of one day. Based on these values, the mean value and standard deviation were calculated from three replicates.

### CFSE-dilution assay

The CFSE dilution assay was performed as previously described with some modifications^93^. When a CFSE-labeled cell divides, its progeny contains half the number of carboxyfluorescein-tagged proteins, hence each cell division can be assessed by measuring the corresponding decrease in cell fluorescence using flow cytometry. For the assay, cells were washed twice with DPBS, and the cell numbers were adjusted to 5 × 10^6^ cells/mL in DPBS. Then, one volume of CFSE solution (5 µM in DPBS) was added to reach a final concentration of 2.5 µM CFSE and 2.5 × 10^6^ cells/mL. After 5 min incubation at RT in the dark, the labeling was quenched by the addition of one volume of FCS. The cells were washed once with DPBS (200*g*, 5 min) and resuspended in the respective conditioned medium. 150,000 CFSE-stained cells were then seeded per well in 12-well plates (cultivation volume: 1 mL). After 24 h of incubation, a freshly prepared particle suspension (1–10 µL, concentration-dependent) was added, and the cells were incubated for another 72 h. Cells incubated without particles under otherwise identical conditions were used as the negative control.

### Statistical analysis

Statistical analysis was performed using Origin software 2019b (Origin, Northampton, MA, USA). All data were tested concerning the homogeneity of variances (Levene test). To investigate differences in MPP interactions with cells and proliferation results, a one-way ANOVA with a Tukey post hoc test was used.

## Supplementary Information

Supplementary Information.
