# Supplementary material for: Noxic effects of polystyrene microparticles on murine macrophages and epithelial cells
Source: Sci Rep. 2021 Aug 3;11:15702. doi: 10.1038/s41598-021-95073-9 (PMC8333329; doi:10.1038/s41598-021-95073-9)
Supplement: Supplementary file 1 — Supplementary Information. [file 41598_2021_95073_MOESM1_ESM.docx]

Noxic Effects of Polystyrene Microparticles on Murine Macrophages and Epithelial Cells

Julia Rudolph*^1^, Matthias Völkl*^2^, Valérie Jérôme^2^, Thomas Scheibel^#1,3,4,5,6^, Ruth Freitag^#2,4^

* shared first author
# co-corresponding authors

**Supplementary Information**

The particle concentration is depending on the number of cells in the used assay, as well as on the total test volume (equation 2),

| $MPP concentration \left( \frac{\mu g}{mL} \right)=$  $\frac{MPP conc. \left( \frac{MPP}{cell} \right)*number of cells}{total test volume \left( mL \right)}*( \rho_{MPP} \left( 1.05\frac{g}{{cm}^{3}} \right)* Particle volume \left( {cm}^{3} \right))$ | (2) |
| --- | --- |

the density of PS-MPP (ρ_MPP_) is 1.05 g/cm^3^. Particle volume can be calculated using the respective particle size

**
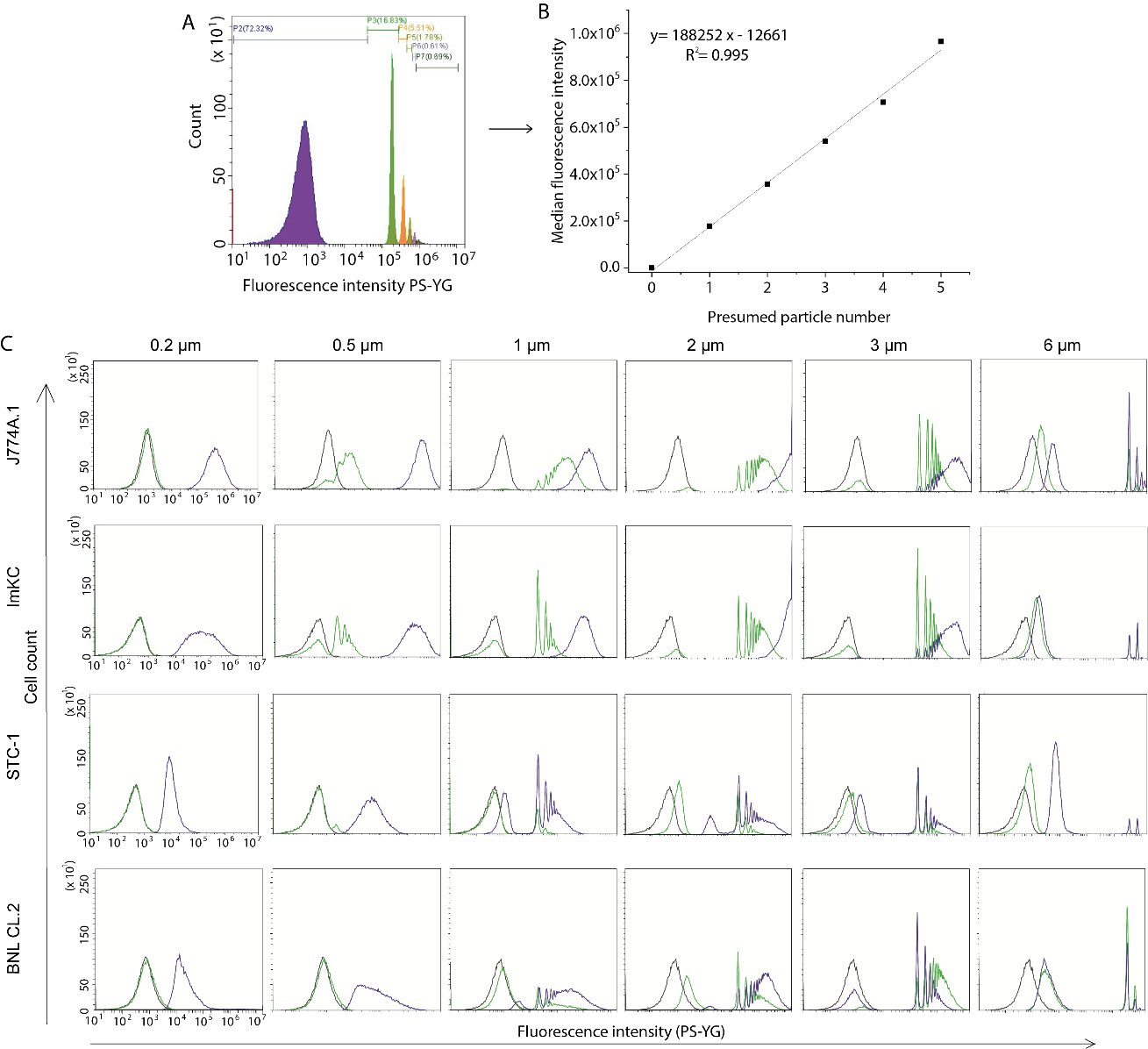
**

**Figure S1. Calculation of particles per cell based on flow cytometry data.**  Different cell populations were gated using the fluorescence intensity of green fluorescent MPPs. (A) The presumed particle number was plotted based on the mean fluorescence intensity of the populations. (B) There is a linear relationship between fluorescence intensity and presumed particle number (R^2^= 0.995). (C) Shown is one replicate of each measurement. Grey: control without particles, green: low MPP concentration, blue: high MPP concentration.


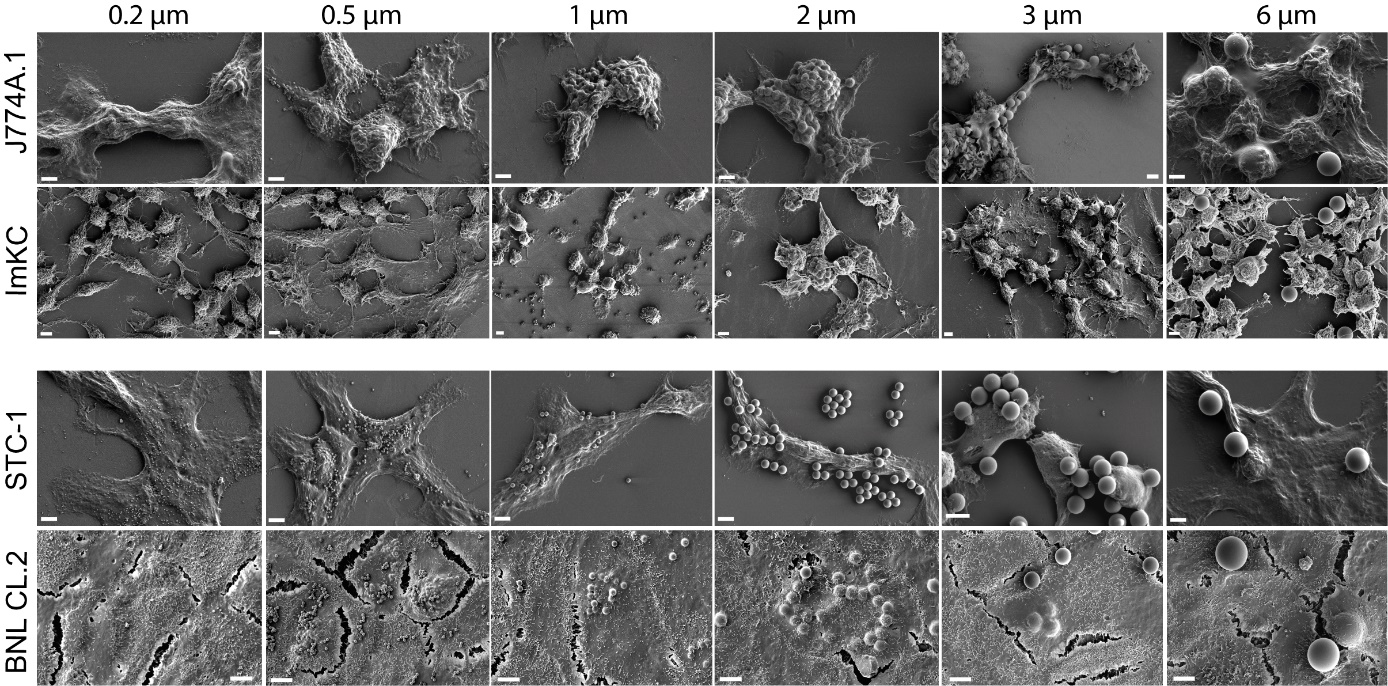


**Figure S2. Size-depenent particle uptake analyzed using SEM.** All cell lines were incubated in the presence of the differently sized PS-MP for 24 hours. Macrophages cells (J774A.1, ImKC) showed ingested particles, which were surrounded by the cell membrane. For epithelial cell lines (STC-1, BNL CL.2), differences were observed in uptake behavior. STC-1 cells did not show particle ingestion, whereas for BNL CL.2 many particles were found surrounded by the cell membrane. Scale bar: 4 µm.


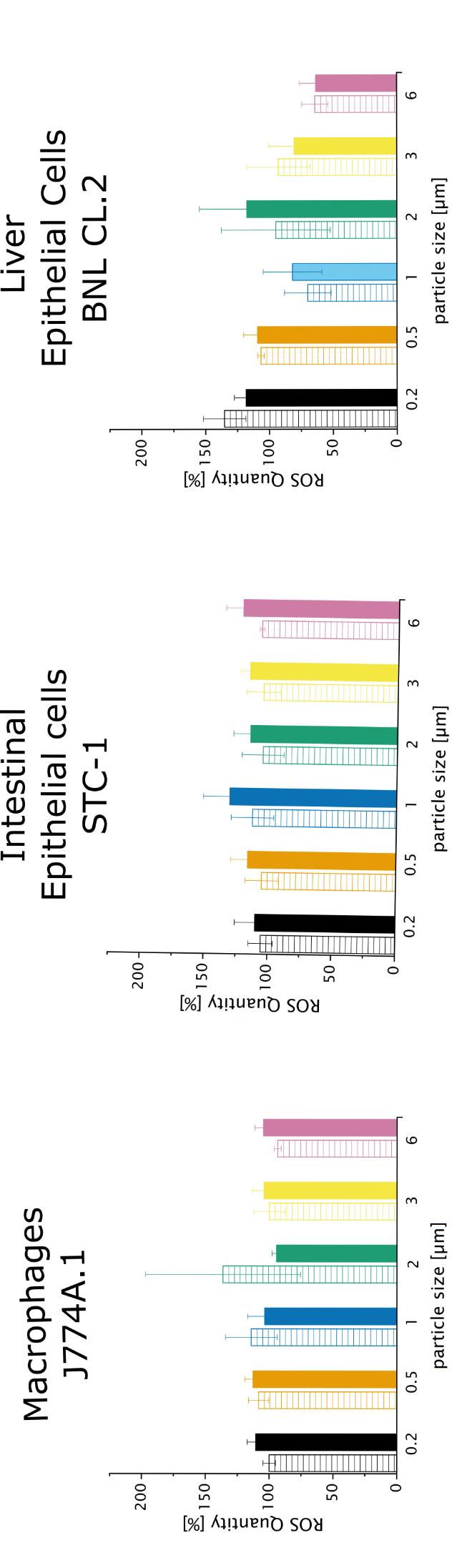


**Figure S3. Appearance of reactive oxygen species upon increasing particle concentrations.** ROS of the whole cell population treated with an increasing particle concentration (striped bars = low concentration; light bar = medium concentration full bars = high concentration). Quantity is represented in relation to a negative control without particles (100 %). Data represent mean ± SD, n = 3.* = p < 0.05, ** = p < 0.01 compared to negative control.


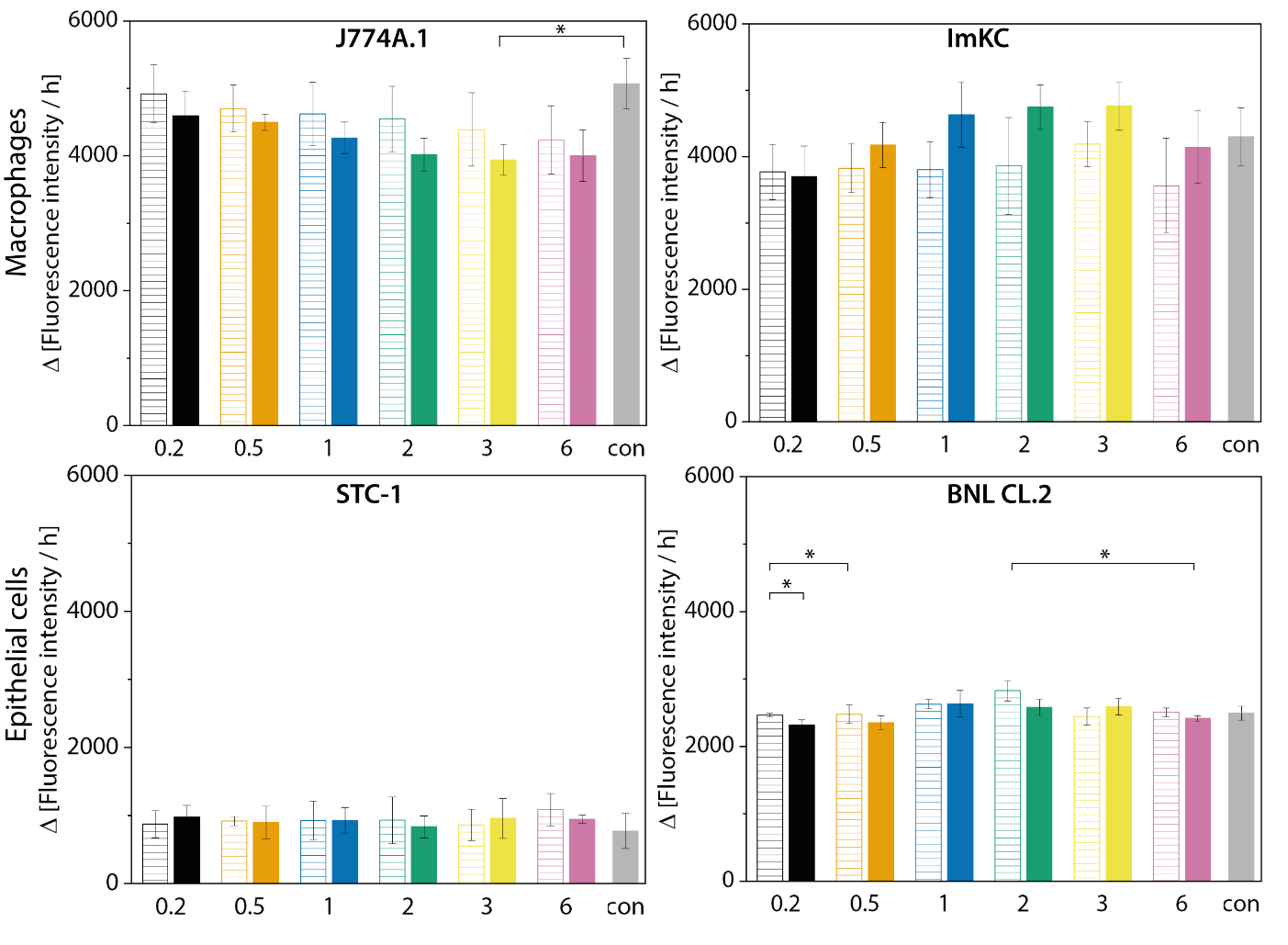
**Figure S4. Cellular proliferation detected by the change in fluorescence intensity per hour (resazurin assay).** The resazurin assay was performed with all cell lines. Two concentrations of all MPP sizes were used (Table S1, low concentration: striped bars, high concentration: filled bars). Con: control cells without MPP. Significant differences were highlighted (*= p < 0.05). Data represent mean ± SD, n = 3.
